# Paradoxical Exception to Island Tameness: Increased Defensiveness in an Insular Population of Rattlesnakes

**DOI:** 10.3390/toxins16030157

**Published:** 2024-03-18

**Authors:** William K. Hayes, Carl E. Person, Gerad A. Fox, Julie L. King, Erick Briggs, Eric C. K. Gren

**Affiliations:** 1 Department of Earth and Biological Sciences, Loma Linda University, Loma Linda, CA 92350, USA; caliginis@yahoo.com (C.E.P.); gfox411@gmail.com (G.A.F.); 2 Animalia Herpetofauna, P.O. Box 63077, Pipe Creek, TX 78063, USA; 3 Catalina Island Conservancy, P.O. Box 2739, Avalon, CA 90704, USA; julielking@hotmail.com; 4 Natural Solutions Wildlife Enterprises, P.O. Box 418, Yucca Valley, CA 92286, USA; erickbriggs@ittybittyoffice.com; 5 Department of Biological Sciences, Southwestern Adventist University, Keene, TX 76059, USA; eric.gren@mso.umt.edu; 6 Bitterroot College, University of Montana, Hamilton, MT 59840, USA; 7 Asclepius Snakebite Foundation, Centennial, CO 80015, USA

**Keywords:** cloacal gland discharge, island syndrome, raptors, rattling, venom expenditure, Viperidae

## Abstract

Island tameness results largely from a lack of natural predators. Because some insular rattlesnake populations lack functional rattles, presumably the consequence of relaxed selection from reduced predation, we hypothesized that the Santa Catalina Island, California, USA, population of the southern Pacific rattlesnake (*Crotalus helleri*, which possesses a functional rattle), would exhibit a decrement in defensive behavior relative to their mainland counterparts. Contrary to our prediction, rattlesnakes from the island not only lacked tameness compared to mainland snakes, but instead exhibited measurably greater levels of defensiveness. Island snakes attempted to bite 4.7 times more frequently as we endeavored to secure them by hand, and required 2.1-fold more time to be pinned and captured. When induced to bite a beaker after being grasped, the island snakes also delivered 2.1-fold greater quantities of venom when controlling for body size. The additional venom resulted from 2.1-fold larger pulses of venom ejected from the fangs. We found no effects of duration in captivity (2–36 months), which suggests an absence of long-term habituation of antipredator behaviors. Breeding bird surveys and Christmas bird counts indicated reduced population densities of avian predators on Catalina compared to the mainland. However, historical estimates confirmed that populations of foxes and introduced mammalian predators (cats and pigs) and antagonists (herbivorous ungulates) substantially exceeded those on the mainland in recent centuries, and therefore best explain the paradoxically exaggerated defensive behaviors exhibited by Catalina’s rattlesnakes. These findings augment our understanding of anthropogenic effects on the behaviors of island animals and underscore how these effects can negatively affect human safety.

## 1. Introduction

Many animals that live on islands exhibit a reduced antipredator response, a phenomenon known as “island tameness”. This phenomenon has been recognized, at least anecdotally, since Darwin’s time [1]. This behavior and associated shifts in the morphology, physiology, ecology, and life history of insular animals are known collectively as “island syndrome” [2,3,4,5]. Although reductions in the morphological, physiological, and behavioral traits associated with antipredator defense may be beneficial in environments with few or no predators [6,7,8], island tameness has rendered many island species highly vulnerable to introduced predators and hunting by humans, resulting in numerous extinctions [9,10,11,12]. Due in large part to this vulnerability, an estimated 95% of bird and mammal extinctions since 1500 Common Era have occurred on islands [13], and insular reptiles have suffered as well [14,15,16,17].

Island tameness results largely from a lack of natural predators [18,19,20,21,22]. In reptiles, behavioral differences between mainland and insular populations have been attributed not only to cognitive deficits in recognizing potential predators [23], but also to hormonal [24,25] and thermoregulatory [26,27] differences underlying the initiation of escape behavior. Antipredator behaviors can also be influenced by experiential effects arising from interactions with humans [28,29,30] or other introduced species [31,32,33,34,35,36]. These influences can complicate the assessment of island tameness, especially when alien species are introduced to an island or an island’s faunal history is unknown.

Venomous animals generally possess a suite of defensive behaviors that include, most prominently, toxin delivery via injection [37]. Rattlesnakes, for example, exhibit a number of antipredator behaviors, including procrypsis, escape, head-elevated coiling, rattling, cloacal gland discharge, bluff strikes, and strikes that include a venomous bite [38,39,40]. Rattlesnakes use rattling as an aposematic acoustic signal [41,42,43,44,45,46] and generally resort to the defensive strike and use of venom only as a measure of last resort [47]. Most individuals of several insular populations of rattlesnakes lack functional rattles, which has been interpreted as a consequence of relaxed selection resulting from isolation and reduced predation [44]. Accordingly, one might expect most island populations of rattlesnakes to exhibit some degree of island tameness.

The southern Pacific rattlesnake (*Crotalus helleri*) offers a compelling opportunity to examine island tameness. The species ranges widely on the southern California mainland, USA, and extends into northern Baja California, Mexico [48], possibly as far south as Bahia San Juanico [49]. Insular populations with dwarf forms exist on two Pacific islands: Santa Catalina Island, California, USA, and Isla Coronado Sur, Baja California, Mexico [46,50]. The population on Isla Coronado Sur is restricted to approximately 1.5 km^2^ of the 3 km^2^ island due to the steep sides of the island [51], whereas the population on Santa Catalina Island ranges throughout much of the 194 km^2^ island. Santa Catalina Island and its rattlesnake population should not be confused with Isla Santa Catalina in the Gulf of California, Mexico, which harbors the rattleless rattlesnake, *C. catalinensis*. Fossil evidence suggests that rattlesnakes formerly existed on two additional California Channel Islands (San Miguel and Santa Rosa Islands) in the late Pleistocene [52,53]. *Crotalus helleri* is part of a larger clade that includes at least five other species (*C. cerberus*, *C. concolor*, *C. lutosus*, *C. oreganus*, and *C. viridis*) and several subspecies [54]. Most rattlesnakes within the clade are highly defensive; when encountered, they often rattle immediately and will strike repeatedly, if necessary, at a persistent threat, especially one that makes physical contact [55,56,57].

In this study, we compared the defensive behaviors of mainland and Santa Catalina Island (hereafter referred to as Catalina) populations of the southern Pacific rattlesnake and inferred a possible cause of the behavioral differences. Although a continental island, along with seven other major Channel Islands off the southern California coast, Catalina has never been physically attached to the mainland and has been separated by a broad channel (currently 32 km) with strong currents [58]. The absence of fossils [59] and genetic analysis preclude the dating of rattlesnake colonization(s) on Catalina. Storm-associated rafting may have occasionally augmented the initial rattlesnake population (snakes have been observed on floating storm debris near the island itself; J. King, pers. obs., and others, pers. comm.), but the population has likely experienced substantial isolation since the Pleistocene, when other rattlesnake populations existed on the northern Channel Islands [52,53]. We therefore hypothesized that Catalina snakes would exhibit behavioral decrements in rattling, cloacal gland discharge, capture difficulty by a human, biting propensity, and venom expenditure. The findings showing that *C. helleri* on Isla Coronado Sur appears to be less risk averse and potentially more explorative than mainland counterparts [60], and that non-venomous gopher snakes on two other Channel Islands exhibit a reduced tail vibration defensive response compared to mainland counterparts [61], support our hypothesis. Nevertheless, both natural and introduced snake predators and antagonists exist on Catalina, and these must be taken into consideration when interpreting differences in behavior that might be detected. Significant threats to the rattlesnakes include ophiophagous (snake-eating) snakes, predatory birds, and all relatively large mammal species [46]. We distinguish between predators, which consume snakes, and antagonists, which may harass and/or kill snakes but do not consume them. Antagonists include herbivorous ungulates (deer and other hoofed animals) that can trample rattlesnakes or even stomp them to death [46]. To better interpret the antipredator behaviors of rattlesnakes, we compared the mainland and island population densities of these other animals that interact with the snakes.

## 2. Results

We obtained data on defensive behavior from 20 mainland snakes and 10 Catalina snakes. However, one snake from each population refused to expulse venom during venom extraction, thereby reducing the sample size for the three measures of venom. Table 1 compares snake size and defensive behaviors between the two populations. The mainland snakes averaged 34% larger in size (based on snout–vent length, SVL) than those from Catalina (*p* ≤ 0.001; Table 1), which required us to control for body size in all analyses.

### 2.1. Snake Rattling

The proportion of rattlesnakes that rattled increased from 50% before pinning for both populations to 90% during capture for the mainland population and 80% for the Catalina population (Table 1). Rattling decreased slightly during venom extraction to 80% and 70% for individuals of the respective populations (Table 1). Logistic regression models indicated no differences between the populations in propensity to rattle in any of the three sequential time periods: before being grasped (captured), during grasping (capture), and during venom extraction (Table 2). Rattling was also independent of snake length and duration in captivity (Table 2).

### 2.2. Snake Cloacal Gland Discharge

Scent gland discharge by rattlesnakes occurred only during grasping. Just three mainland snakes (15%) and one Catalina snake (10%) exhibited this behavior (Table 1), which was also independent of snake length and duration in captivity (Table 2).

### 2.3. Snake Bite Attempts, Latency to Capture, and Venom Expulsion

Catalina snakes attempted to bite 4.7-fold more often than mainland snakes (*p* = 0.003; Table 1). Logistic regression indicated that bite attempts were independent of body size and duration in captivity (Table 2). A multiple analysis of covariance (MANCOVA) model comparing the combined dependent variables of latency to capture, venom expended, the number of bilateral venom pulses (sum of pulses observed from right and left fangs, divided by two; see Methods), and venom per bite (bite = two pulses) yielded significant effects for population (Wilks’ λ = 0.44, *F*_4,21_ = 6.77, *p* = 0.001, adjusted multivariate partial η^2^ = 0.53) snake length (Wilks’ λ = 0.59, *F*_4,21_ = 3.65, *p* = 0.021, adjusted multivariate partial η^2^ = 0.38). Duration in captivity had no effect (Wilks’ λ = 0.91, *F*_4,21_ = 0.54, *p* = 0.71, adjusted multivariate partial η^2^ = 0.09).

**Figure 1:**
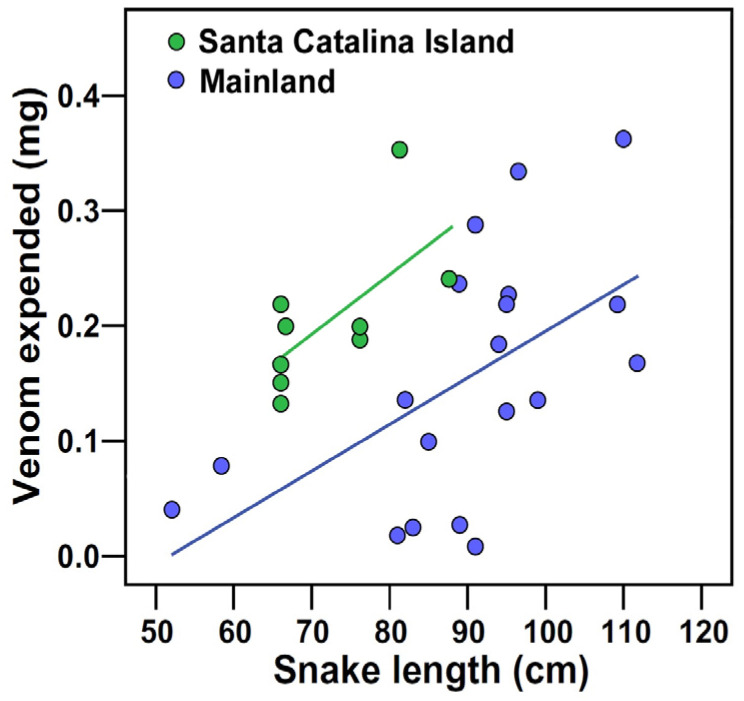
Wet mass of venom expended during venom extractions of mainland (*N* = 19) and Santa Catalina Island (*N* = 9) populations of the southern Pacific rattlesnake (*Crotalus helleri*). Island specimens delivered significantly more venom than those from the mainland.

Post-hoc univariate analysis of covariance (ANCOVA) models revealed that some dependent variables contributed more to the MANCOVA model outcome than others. Latency to capture the snake (Table 1) differed significantly between populations (*p* < 0.001) and was independent of snake length and duration in captivity (Table 3). Catalina snakes required 2.1-fold more time to be captured than the mainland snakes (Table 1). Venom expenditure (Table 1) differed between populations (*p* = 0.002), but was positively associated with snake length (*p* = 0.001; Table 3; Figure 1). When controlling for snake length, the island snakes delivered 2.1-fold more venom when biting (estimated marginal means at 85 cm for SVL; Table 1). The number of bilateral venom pulses was similar for the two populations (Table 1) and independent of snake size and duration of captivity (Table 3). However, the mass of venom per bite (Table 1) differed between populations (*p* = 0.012) and was positively associated with snake length (*p* = 0.002; Table 3, Figure 1). When controlling for snake length, the island snakes expulsed 2.1-fold more venom per bilateral pulse (estimated marginal means at 85 cm for SVL; Table 1).

### 2.4. Relative Head Size

A MANCOVA model including both head length and head width as the combined measure of head size revealed no differences between populations (Wilks’ λ = 1.00, *F*_2,58_ = 0.11, *p* = 0.89, multivariate partial η^2^ = 0.00) or sexes (Wilks’ λ = 0.93, *F*_2,58_ = 2.33, *p* = 0.11, multivariate partial η^2^ = 0.07). However, relative head size declined with an increase in body length (Wilks’ λ = 0.11, *F*_2,58_ = 226.83, *p* < 0.001, multivariate partial η^2^ = 0.89).

### 2.5. Mainland versus Island Raptor Abundance

**Figure 2:**
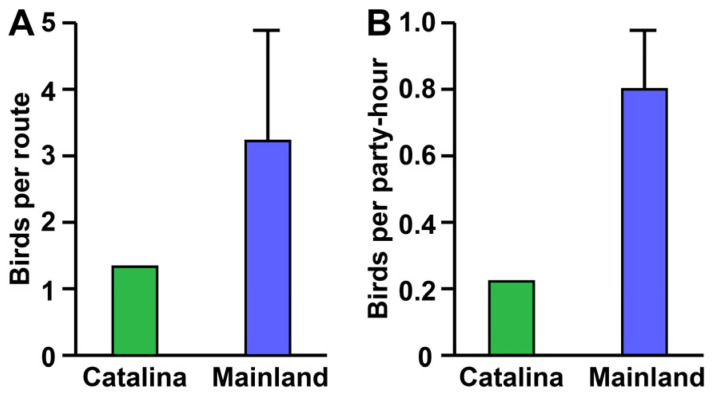
Raptor densities on Catalina Island and the adjacent mainland of southern California. (**A**) Spring breeding bird surveys. (**B**) Christmas bird counts.

During breeding bird surveys (BBBs) and Christmas bird counts (CBCs), only one raptor predator of snakes was recorded on Catalina (*Buteo jamaicensis*), whereas three bred (*Aquila chrysaetos*, *B*. *jamaicensis*, and *B. lineatus*) on the mainland and were joined by six additional species during the winter (*B. albonotatus*, *B. lagopus*, *B. platypterus*, *B. regalis*, *B. swainsoni*, and *Parabuteo unicinctus*). Raptor density (mean ± 1 S.D.) was significantly less on Catalina compared to the mainland during both the breeding season (BBSs: 1.29 and 4.25 ± 2.89 birds per survey route, respectively; one-sample *t*_24_ = 5.11, *p* < 0.001, 95% C.I. of difference = 1.77–4.16, Cohen’s *d* = 1.02) and winter (CBCs: 0.23 and 0.80 ± 0.33 birds per party hour, respectively; one-sample *t*_11_ = 5.92, *p* < 0.001, 95% C.I. of difference = 0.36–0.78, Cohen’s *d* = 1.71; Figure 2).

### 2.6. Mainland versus Island Mammal Abundance

Despite the low diversity of mammalian carnivores and omnivores on Catalina, their collective density (feral cats [*Felis catus*]: 3.1–3.9/km^2^; foxes [*Urocyon littoralis catalinae*]: 5.7–6.9/km^2^; feral pigs [*Sus scrofa*]: 6.5–10.5/km^2^; Table 4) has, in recent history, probably exceeded that of natural areas on the mainland. Camera-trap studies across coastal California suggest that among mammalian carnivores, coyote (*Canis latrans*; 0.3–0.4/km^2^ in natural areas; [62]) and bobcat (*Lynx rufus*; 0.25–1.5/km^2^; [63]) populations may be the most dense on the mainland [64]. Estimates from individual locations can nevertheless be much higher for some of the other carnivores [65,66], which include the American badger (*Taxidea laxus*; 0.39–5.0/km^2^), gray fox (*Urocyon cinereoargenteus*; 0.4–10.0/km^2^), long-tailed weasel (*Mustela frenata*; 0.38–38/km^2^), mountain lion (*Puma concolor*; 0.005–0.048/km^2^), raccoon (*Procyon lotor*; 2.3–20.0/km^2^), striped skunk (*Mephitis mephitis*; 1.8–4.8/km^2^), Virginia opossum (*Didelphis virginiana*; 2–116/km^2^), and western spotted skunk (*Spilogale gracilis*; 8.8–40/km^2^). Some of the smaller mainland mesopredators (e.g., weasel, raccoon, skunks, and opossum) may pose little threat to rattlesnakes [46], though the snakes may still respond defensively during encounters. Domestic dogs (*Canis lupus familiaris*) were probably common at one time on the island [67], but because there has been no substantial feral population in recent history, we have no estimates of population size. Feral cats and pigs also occur in scattered locations on the mainland, but these island densities far exceed those on the nearby mainland where, for example, areas with 3–4 pigs/km^2^ would be exceptional [68].

Perhaps more importantly, population densities of potential antagonists, the herbivorous ungulates, have long been exceptional on Catalina (Table 4). Ungulate densities on the island in the past two centuries have generally been in the many tens to hundreds of individuals per km^2^, compared to mainland (statewide) densities of deer of 4–9/km^2^ [76,89], with concentrations of more than 20/km^2^ in relatively small areas [90,91]. Much smaller numbers of bighorn sheep (*Ovis canadensis*) remain on the mainland [92], but their population and that of the regionally extirpated pronghorn (*Antilocapra americana* [93]) were undoubtedly greater in prior centuries (no estimates exist).

Humans are both antagonists and predators of rattlesnakes. Catalina historically supported an estimated 2000–3000 indigenous North Americans [94]. The island currently supports a human population of 3460 (U.S. Census Bureau estimate for 2020) and close to 1 million visitors annually (Catalina Island Chamber of Commerce and Visitors Bureau, unpublished data). Because most human activity has been on the island’s periphery, comparisons of population density to that of the mainland population lack relevance.

## 3. Discussion

The results of this study unambiguously reveal substantially greater defensiveness in Catalina rattlesnakes compared to mainland rattlesnakes. Of the nine measures of antipredator behavior that we quantified, four differed significantly between the two populations. Snakes from the island population attempted to bite 4.7-fold more frequently as we endeavored to secure them by hand, and as a result, they required 2.1-fold more time to be pinned and captured. When induced to bite a membrane-covered beaker after being grasped, the island snakes ejected a similar number of venom pulses, but delivered 2.1-fold greater quantities of venom. The additional venom resulted from 2.1-fold larger pulses of venom being expulsed from the fangs during biting. Importantly, we found no effect of duration in captivity (ranging from 2–36 months) on any aspect of defensive behavior, which suggests an absence of long-term habituation and a negligible effect of captive conditions on the behaviors that we quantified. In the sections that follow, we discuss the relevance of each of these behavioral differences and possible causes, particularly in relation to potential avian and mammalian predators and antagonists.

### 3.1. Rattling

The rattle of rattlesnakes most likely evolved under pressure from predation to become an aposematic acoustic signal [41,42,44,45,46,95]. Several island populations in the Gulf of California (genus *Crotalus* [44]) and a mainland species of rattlesnake (*Sistrurus miliarius* [95]) are largely lacking functional rattles, which has been attributed to relaxed selection on the interlocking lobes and grooves of adjacent segments, thereby resulting in loss of the older segment during ecdysis. The theory of relaxed selection, however, is problematic because morphological vestigialization (loss) of the rattle has been decoupled from the behavioral and physiological expression of rattling, which still persists in island populations of rattlesnakes lacking rattles [44]. We expected the Catalina specimens to rattle less frequently, in part due to comments that we heard from those who live on the island and from those who have captured them. However, our results provide evidence of yet another island population showing no decrement in rattling behavior.

### 3.2. Cloacal Gland Discharge

Snakes occasionally discharge their cloacal gland in a defensive context, though the function remains unclear. Often coupled with tail rubbing, presumably to spread the exudate [96], cloacal discharges serve as a noxious defense that may render the snake unappetizing to a potential predator [46,97]. Cloacal discharge may disrupt or delay the predatory sequence, or serve as an emetic [98], but while it may deter potential feline predators [99], it apparently has a negligible effect on potential canine predators [100]. It may also serve as an alarm pheromone for intraspecific communication [38], particularly among aggregated gravid females [96]. In garter snakes (genus *Thamnophis*), cloacal discharge frequency was consistent across variations in predation pressure, environmental conditions, and snake body size; however, musking propensity differed between the two species examined, as it occurred more frequently in females than males and was positively associated with body condition [101]. We noted no difference in propensity to discharge the scent gland between mainland and island rattlesnakes, and individuals from neither population relied often on this strategy (10–15% of trials). Studies of defensive behavior in another crotaline snake, the Cottonmouth (*Agkistrodon piscivorus*), similarly reported infrequent use of this tactic [102,103,104].

### 3.3. Biting and Venom Expenditure

Rattlesnakes and venomous snakes in general depend heavily on their venomous bite to defend themselves. Reliance on biting (venom injection) for defense can vary with the snake’s body size, internal state (e.g., body temperature, recent ingestion, or pregnancy), microhabitat, proximity to resources (e.g., refugia or recently born young), environmental conditions (e.g., temperature, cloud cover, and season), perception of threat intensity, accumulated experience, and other factors [47,102,103,104,105,106,107,108,109,110]. Under the conditions tested, Catalina snakes attempted to bite more frequently when we tried to pin and grasp the snake, which resulted in a greater latency to capture the snake. We consider our actions toward the snake reasonably comparable to those of a natural predator which might seek to subdue a snake. Although venomous snake bites may be ineffective against some enemies, particularly those that possess physiological resistance to the venom [111,112], the bites can still be painful and fatal bites are sometimes delivered [46,113,114].

When grasped by the head and induced to bite a target, the Catalina snakes expulsed more venom than mainland snakes when body size was controlled for (Figure 1). The greater mass of venom injected by Catalina snakes resulted from the delivery of more venom per pulse rather than by increasing the number of pulses through multiple bites. These unexpected findings could result from three non-mutually exclusive possibilities: (1) differences in relative head size, and therefore quantities of venom available to be injected; (2) a behavioral response corresponding to the level of defensiveness; or (3) selection favoring the delivery of larger venom doses to compensate for the comparatively smaller body size of the island snakes. The quantity of venom that rattlesnakes deliver in a bite corresponds strongly to body size, and therefore head and venom gland size [55,115], but morphological comparisons revealed no difference in relative head size between the insular and mainland populations; thus, the first explanation based on differences in relative head size seems unlikely. Rattlesnakes make decisions about how much venom to inject and appear to do so in a threat-sensitive manner [55,57], so the second explanation invoking behavioral modulation of venom delivery seems reasonable. Two additional measures of defensive behavior were similarly elevated in the island snakes (the number of bite attempts and latency to capture the snake), supporting our interpretation that venom expenditure corresponds to the levels of defensiveness. Further investigation is necessary to assess the third possibility, that the island snakes deliver larger quantities of venom during defensive bites because of their smaller body size.

### 3.4. Possible Causes of Increased Defensiveness

After we determined that rattlesnakes from Catalina exhibit paradoxically heightened levels of defensiveness, we turned our attention to the possible causes. Historically, low levels of predation pressure on the island can be inferred, for example, from the former existence of a flightless duck on the island [116]. Although antipredator behaviors of rattlesnakes [44,117] and other vipers [118] may persist on islands under conditions of relaxed selection, possibly due to the presence of at least some of the mainland predators (the multipredator hypothesis [119]), something has caused an exaggerated antipredator response on Catalina relative to the mainland. Among the naturally occurring candidates responsible for the exaggerated defensiveness in Catalina rattlesnakes, we can immediately rule out ophiophagous snakes, which are likely to be no more common than on the mainland and elicit antipredator responses (head-hiding and body-bridging [120,121]) that are very different to those we observed and measured. We can also rule out avian predators, since our analyses of BBS and CBC data confirm that the only significant raptor predators exist at much lower densities on Catalina than on the mainland. We are left, then, with the most likely explanation that exceptionally high densities of introduced mammalian predators and antagonists (Table 4) are responsible for the increased defensive behavior in Catalina rattlesnakes. Current densities of these animals on Catalina substantially exceed those of the mainland. Although we do not know historic densities of ungulates on the mainland, they were unlikely to have approached those in the past two centuries on Catalina, which have substantially denuded the vegetation and at times exceeded carrying capacity [76,84,122,123,124].

Humans are believed to have arrived in the Channel Islands approximately 13,000 years ago [125] and to have populated Catalina roughly 8000 years ago [94]. Although human interaction as a contributing factor to snake behavior change seems plausible, as has been postulated for the elevated wariness or defensiveness of Orange-throated Whiptail Lizards (*Aspidoscelis hyperythra*) on islands in the Sea of Cortez [29] and of Aegean Wall Lizards (*Podarcis erhardii*) on islands in the Mediterranean Sea [32], we lack information on how snake–human interactions might differ between the island and the mainland. However, humans brought with them to the Channel Islands a wealth of mammalian predators and antagonists (Table 4) whose potential impacts on rattlesnakes are well known [46]. As we have documented, the collective population density of these mammals has likely exceeded that of the mainland in recent centuries and up to the present day.

Whether the population differences in behavioral defensiveness resulted from underlying genetics or phenotypic plasticity remains unclear. Evidence for both genetic and experiential mechanisms of island tameness exists for other snakes [105,126,127]. Elevated wariness and defensiveness in human-hunted populations of the Japanese mamushi (*Gloydius blomhoffii*), and demonstrated heritability of these behaviors [30], suggests the capacity for rapid evolution of antipredator behaviors in viperid snakes. Because the snakes that we subjected to behavioral tests were all adults acquired from the wild, prior experiences of the snakes may have contributed to the differences that we measured. However, we found a negligibly small effect of duration in captivity on defensiveness, suggesting that the behaviors that we analyzed were minimally affected by a history of infrequent disturbance and handling. We therefore suggest that Catalina snakes, in recent centuries, have interacted with introduced mammals in ways that have enhanced their innate defensiveness. Because behavior can expose organisms to or protect them from novel selection pressures, behavior often evolves faster than other traits [128,129,130]. Thus, behavior has been called the “pacemaker of evolution,” as it can influence the evolution of morphological, physiological, life history, and other traits. In the curious case of insular rattlesnakes, enhanced defensiveness (demonstrated in this study) may evolve more rapidly in a predator- and antagonist-rich environment than relaxed defensiveness in a relatively predator- and antagonist-free environment [44]. Yet under relaxed selection with few predators, morphological changes in the rattle have appeared to occur more rapidly than behavioral changes associated with rattle use [44], a finding that seems counterintuitive and invites further investigation.

One additional potential cause for the heightened defensiveness of Catalina rattlesnakes merits consideration. These snakes, which average smaller in size than those on the mainland, frequently prey upon several rodent species [131] that average larger in body size than those on the mainland: the native Catalina California ground squirrel (*Otospermophilus beecheyi nesioticus*) [132] and the native or possibly introduced deer mouse (*Peromyscus maniculatus catalinae*) [133,134]. Feeding on relatively large rodents with formidable teeth and claws poses tangible risks to snakes, including injury and even death [135,136]. To minimize the risks of retaliatory injury, rattlesnakes and other vipers have evolved unique strategies to acquire dangerous prey, including the release of prey immediately after the envenomating bite, followed by chemosensory searching to relocate their meal, which often travels several meters or more before succumbing to the venom [137,138]. This size differential between predator and prey may have resulted in selection for relatively larger quantities of venom injected during bites for the island snakes, and perhaps even heightened defensiveness to protect against retaliation by envenomated prey or by adult rodents defending their pups. Further investigation is needed to examine this possibility. Other explanations for increased rattlesnake defensiveness exist that we cannot speculate on, including selection for niche specialization in general, mate competition, and pigmentation (melanin production and aggression are physiologically linked via the melanocortin system) [139]. Rattlesnake defensiveness might also vary with environmental and/or social attributes [140].

## 4. Conclusions

Island ecosystems have provided a wealth of important insights into numerous ecological and behavioral phenomena. Among the various expressions of island syndrome, island tameness as a behavioral trait might be the first to respond to environmental change. Here, we have shown that an insular population of southern Pacific rattlesnakes exhibits paradoxically increased defensiveness relative to mainland conspecifics, and we have attributed it to human introductions of non-native mammalian predators and antagonists. Although island tameness is widespread among animals, including reptiles [4,19,20,35,36,141], our study adds to a limited but growing body of evidence that island tameness may be reversed when environments undergo anthropogenic changes [29,30,31,32]. Our findings further underscore the need to shift attention from decrements in defensiveness to increments in defensiveness in island animals, including comparisons of their evolutionary rates.

Anthropogenic changes in animal behavior can have important implications for human health and can result from three major routes. First, through selective breeding, we have induced behavioral changes in companion and consumption animals that have been both beneficial and detrimental to human emotional and nutritional health [142,143]. Second, our impacts on the environment have altered the behaviors of certain disease hosts or vectors in ways that have enhanced disease transmission [144]. Third, our impacts on the environment have also exacerbated food stress, habituation, and direct conflict in large predators, herbivores, and primates, resulting in increased attacks on humans [145,146]. Rattlesnakes differ from animals of the third category in that they only bite defensively as a measure of last resort. Indeed, deliberate provocation is associated with nearly half (45.8%) of human envenomations by mainland southern California rattlesnakes [147]. Because the rattlesnakes on Catalina possess a dangerous venom [148,149], their tendency to bite more readily and inject larger quantities of venom increases the risks associated with humans interacting with snakes from this population and potentially suffering severe or even fatal envenomation. Thus, by introducing mammalian predators and antagonists to Catalina, humans have made encounters with the rattlesnakes more dangerous, a fact that should be appreciated by those who currently oppose the removal of introduced deer from Catalina [150]. We suspect the same may be true for islands elsewhere that support both venomous snakes and high densities of introduced snake predators and antagonists.

## 5. Materials and Methods

### 5.1. Snake Subjects

We obtained data from two groups of snakes. We initially collected morphological data, as part of a separate study, from 46 snakes from the mainland (30–112 cm SVL; 23 males and 23 females) and 18 snakes from Catalina (27–95 cm SVL; 16 males and 2 females). These data, obtained by digital calipers, included measures (to nearest 0.1 mm) of head length from the rostral to the inflection of the neck, and head width at the widest point. We later subjected those specimens that remained in our collection and some additional mainland snakes to behavioral tests, which included 20 snakes from the mainland (52–117 cm SVL; 14 males and 6 females; 2–36 months in captivity) and 10 snakes from Catalina (64–88 cm SVL; 9 males and 1 female; 12–24 months in captivity). Mainland snakes were obtained from six southern California counties, including Los Angeles (*N* = 43 for morphology, 15 for behavior), Riverside (15, 2), San Bernardino (14, 3), San Diego (9, 8), Santa Barbara (2, 0), Ventura (0, 1), and unknown (0, 1). Snakes were housed in terrariums of varying size (968–5547 cm^2^ floor space) with either newspaper or pine shavings as a substrate, and maintained in rooms at 24–28 °C. Snakes were regularly fed mice or small rats every 2–3 weeks, and water was provided ad libitum in a small container. We withheld food from all subjects for 22 d prior to each behavioral trial.

### 5.2. Snake Behavioral Trials

A trial began when one investigator (always CEP) removed the snake from its home cage using a snake hook and placed it on a piece of carpet on the floor. Another investigator (always WKH, who was “blind” to which population the snake was from except for the largest snakes, which were always from the mainland) pinned the snake with a snake hook, leaned over to grasp the snake by the head and neck, and then induced the snake to bite a Parafilm-covered beaker secured to a ring stand [151]. We videotaped each venom extraction using a Logitech Orbit AS web cam (Logitech, Lausanne, Switzerland; 30 fields/s). While wearing gloves, we subsequently transferred the venom to a pre-weighed 1.5 μL microcentrifuge tube, which was weighed again to determine the wet mass of the venom (nearest 0.01 mg) on an electronic analytical balance (model R-160P; Sartorius Research, Bohemia, NY, USA).

While removing each snake from its cage, we noted all attempts by the snake to bite and any tail rattling. Once the snake was on the carpet, we recorded the time required (nearest 1 s) to pin the snake and grasp it, and noted whether the snake ejected musk from its cloacal glands and again any bite attempts and rattling. During venom extraction, we also recorded the occurrence of rattling and musk ejection. During field-by-field video review of the venom extractions, we observed that snakes sometimes delivered unilateral bites (a venom pulse from a single fang only) in addition to bilateral bites (a venom pulse from both fangs). We recorded the number of venom pulses corresponding to a bilateral bite (sum of pulses observed from the right and left fangs, divided by two; [152]). We then computed venom expended (wet mass, nearest 0.01 mg) per bilateral venom pulse or bite [56].

### 5.3. Populations of Snake Predators and Antagonists

We used existing databases to compare the relative abundance of potential snake predators and antagonists on Catalina and the mainland. Relevant population data are lacking for reptile predators, but ample data exist for birds and mammals.

Among reptiles, two ophiophagous snakes occur on Catalina: *Diadophis punctatus* and *Lampropeltis californiae*. Their relative abundance is unlikely to match that of the larger guild of six ophiophagous snakes on the mainland: *Coluber constrictor, Coluber flagellum, Coluber lateralis*, *D. punctatus*, *L. californiae*, and *Lichanura trivirgata*. More importantly, rattlesnakes exhibit very distinctive antipredator responses to ophiophagous snakes (e.g., head-hiding and body-bridging) that were neither observed nor measured in this study [120,121]. Thus, we can rule out predatory snakes as an explanation for any differences in the defensive behaviors that we measured between mainland and island rattlesnakes. 

Among birds, the only southern California taxa likely to exert significant predatory pressure on rattlesnakes are the greater roadrunner (*Geococcyx californianus*) and an assortment of raptors, including the golden eagle (*A. chrysaetos*), hawks of the genera *Buteo* and *Parabuteo*, and the great horned owl (*Bubo virginianus*) [46]. Of these, only the hawks reside on Catalina. We obtained the relative population density of island and mainland raptors (golden eagles and hawks of the genera *Buteo* and *Parabuteo*) from breeding bird survey (http://www.mbr-pwrc.usgs.gov/bbs/bbs.html; accessed on 11 February 2015) and Christmas bird count (http://netapp.audubon.org/CBCObservation/Historical/ResultsByCount.aspx#; accessed on 4 February 2015) data. Standardized BBS protocols record all birds detected within 3 min and 0.4 km of 50 points spaced at 0.8 km intervals along a 39 km route during peak breeding season (May–June). We compared BBS mean annual raptor counts from Catalina (conducted for nine years during the period 1988–2013) and all 25 mainland locations from Los Angeles, Orange, Riverside, San Bernardino, San Diego, Santa Barbara, and Ventura Counties (each conducted from 9 to 36 years during the period 1966–2013). The CBCs comprise single-day counts by groups of birders within a 24 km radius during the winter (December–January). We compared CBC mean raptor counts per party hour from Catalina (54–73 party hours for each of the six years of data collection during the period 2000–2006) and 12 arbitrarily selected mainland locations from the same aforementioned counties (8–444 party hours for each of 5–14 years during the period 2000–2013). No data were available for comparing raptor populations during spring and fall migration.

Among mammals, the diversity of native snake predators and antagonists is clearly depauperate on Catalina compared to the mainland [153]. Only the endemic Santa Catalina Island fox (*U. littoralis catalinae*) may be native, though some evidence suggests that humans introduced it from the northern Channel Islands during the middle Holocene [154]. Foxes on the island rarely prey upon snakes [155]; anecdotal reports and photographs have been shared on social media, though gray foxes (*U. cinereoargenteus*) on the mainland occasionally do so [46], and rattlesnakes might still react defensively during encounters. Three additional predators have been introduced to Catalina, all of which occasionally prey upon snakes [46]: domestic dogs (*C. lupus familiaris*), which accompanied the first human settlers and were likely common at one time [67], though they no longer comprise a feral population; domestic cats (*F. catus*), which exist as a substantial feral population (Table 4); and feral pigs (*S. scrofa*), which are omnivorous ungulates that were abundant prior to their recent extirpation in 2004 (Table 4). Six species of herbivorous ungulates have also been introduced, some of which have been extirpated in recent decades (Table 4). For mammalian predators and antagonists, we compiled data on the dates of introduction for island populations and population density estimates of both mainland and island populations from the published literature. We gleaned additional data from unpublished reports submitted to the Catalina Conservancy and to the Santa Cruz Island Foundation. Humans as both predators and antagonists may have affected to some extent the behavior of mainland and/or island snakes, but we lack meaningful comparative data to draw inferences. Nevertheless, we summarize our understanding of human populations on Catalina.

### 5.4. Analyses

We conducted all statistical analyses using SSPS 13.0 for Windows (Statistical Package for the Social Sciences, Inc., Chicago, IL, USA), with alpha set at 0.05. We rank-transformed all quantitative variables (SVL, time in captivity, latency to capture snake, mass of venom, number of bites, and venom per bite) for the behavioral analyses to conform to parametric assumptions of normality and homoscedasticity. No transformations were needed for the analyses of snake morphology and raptor density. We did not apply Bonferroni adjustments to control for experiment-wise error, because doing so overemphasizes the importance of null hypothesis testing when effect size is more meaningful, and unacceptably increases the probability of making type II errors [156].

For rattlesnake behaviors, we analyzed the dichotomous variables (rattle before grasping, rattle during grasping, rattle during venom extraction, cloacal gland discharge, and attempted to bite) using binomial logistic regression [157], which allowed us to control for body size (SVL) and duration in captivity (months) while comparing behaviors between the two populations. We subjected the continuous variables (latency to capture, venom expended, bilateral venom pulses, and venom per bite) to general linear models, beginning with a MANCOVA model [157] that tested the effects of population (between-subjects factor), SVL (covariate), and duration in captivity (covariate) on the combined dependent variables of latency to capture, mass of venom, number of bites, and venom per bite. We then conducted follow-up univariate ANCOVA models [157] for each dependent variable using the same independent variables (population, SVL, and duration in captivity). We included SVL as a covariate because defensive behaviors and their habituation in captivity can vary with snake size [40,104], and the mass of venom expended by snakes is strongly affected by body size [115]. We also included duration in captivity as a covariate to control for potential habituation or other effects, though the results were identical when we excluded it. To test the ANCOVA assumption of homogeneity of regression slopes, we first tested each model with the inclusion of the interactions between population and the two covariates, and then removed these terms from the final model since no significant interactions existed.

To examine relative head size of the two snake populations, we used another MANCOVA model that included head length and head width as the combined dependent variable, sex and population as independent variables, and SVL as a cofactor. Because relationships were linear and the data met parametric assumptions, we applied no data transformations. We omitted the interactions of population and sex with SVL from the final model because we met the assumption of homogeneity of regression slopes.

For mainland versus island comparisons of raptor abundance, we used separate one-sample *t*-tests [158] for the BBS and the CBC data. Each test compared the mean for all mainland BBSs or CBCs to the corresponding values for Catalina.

We further computed effect sizes for all tests, which are independent of sample size (in contrast to statistical significance), biologically more meaningful, and can be more readily compared among different data sets and different studies [159]. For one-sample *t*-tests, we computed Cohen’s *d*, with values of 0.2, 0.5, and 0.8 corresponding loosely to small, medium, and large effects, respectively [160]. For logistic regression, we calculated odds ratios, which indicate the probability of a switch from one binary state to another with each unit of change in the independent variable(s). Values smaller or larger than 1.0 correspond to increasingly less likely or more likely events, respectively [157]. For the general linear models, we computed multivariate partial (for MANCOVA) or partial (for ANCOVA) eta-squared (η^2^), with values of ~0.01, ~0.06, and ≥0.14 loosely deemed small, moderate, and large effects, respectively [160]. Because η^2^ is upward-biased [161], the η^2^ values for main effects summed to >1.0 in the MANCOVA model for behavior; accordingly, we adjusted the values by dividing each η^2^ value by the sum of all η^2^ values [162].

## Figures and Tables

**Table 1:** Body size and antipredator behaviors (mean ± 1 S.E.) exhibited during venom extractions of mainland (*N* = 20) and Santa Catalina Island (*N* = 10), California, USA, populations of the southern Pacific rattlesnake (*Crotalus helleri*).

Measure	Mainland (*N* = 20)	Catalina Island (*N* = 10)
Snout–vent length (cm)	91 ± 4	72 ± 3
Rattle before grasping (%)	50.0	50.0
Rattle during grasping (%)	90.0	80.0
Rattle during extraction (%)	80.0	70.0
Cloacal gland discharge (%)	15.0	10.0
Attempted to bite (%)	15.0	70.0
Latency to capture (s)	7.7 ± 1.0	15.8 ± 2.2
Venom expended (mg wet mass) ^a,b^	0.13 ± 0.02	0.27 ± 0.03
Bilateral venom pulses (number) ^a,c^	2.0 ± 0.3	2.5 ± 0.2
Venom per bite (mg) ^a,b^	0.07 ± 0.01	0.15 ± 0.02

^a^ *N* = 19 (mainland) and 9 (Catalina); one snake in each population refused to deliver venom. ^b^ Estimated marginal means (corrected for body size) are at 85 cm for snout–vent length. ^c^ Venom pulses corresponded to a bilateral bite (i.e., sum of pulses observed from right and left fangs, divided by two); thus, a unilateral bite (pulse from a single fang) counted as half a bite.

**Table 2:** Logistic regression models for dichotomous antipredator behaviors of mainland (*N* = 20) and Santa Catalina Island (*N* = 10), California, USA, populations of the southern Pacific rattlesnake (*Crotalus helleri*).

Predictors ^a^	B	SE	Wald	*p*-Value	Odds Ratio (95% C.I.)
Rattle before grasping (χ^2^_3_ = 2.55, *p* = 0.47, Nagelkerke *R*^2^ = 0.11, 56.7% predicted correctly)					
Population	1.08	1.24	0.93	0.336	2.95 (0.33–26.70)
Snake length	−0.09	0.07	2.05	0.153	0.91 (0.80–1.04)
Duration captivity	0.002	0.05	0.00	0.961	1.00 (0.91–1.10)
Rattle during grasping (χ^2^_3_ = 2.51, *p* = 0.47, Nagelkerke *R*^2^ = 0.15, 86.7% predicted correctly)					
Population	2.50	2.16	1.33	0.248	12.12 (0.18–838.97)
Snake length	−0.12	0.12	0.94	0.333	0.89 (0.70–1.13)
Duration captivity	−0.05	0.08	0.42	0.518	0.95 (0.82–1.11)
Rattle during extraction (χ^2^_3_ = 1.11, *p* = 0.78, Nagelkerke *R*^2^ = 0.06, 76.7% predicted correctly)					
Population	0.30	1.18	0.06	0.800	1.35 (0.13–13.51)
Snake length	0.02	0.07	0.10	0.754	1.02 (0.89–1.17)
Duration captivity	−0.05	0.06	0.70	0.402	0.95 (0.85–1.07)
Cloacal gland discharge (χ^2^_3_ = 1.33, *p* = 0.72, Nagelkerke *R*^2^ = 0.08, 86.7% predicted correctly)					
Population	−0.60	1.74	0.12	0.729	0.55 (0.18–16.65)
Snake length	0.08	010	0.79	0.374	1.09 (0.90–1.31)
Duration captivity	−0.05	0.07	0.56	0.456	0.95 (0.83–1.09)
Attempted to bite (χ^2^_3_ = 10.55, *p* = 0.014, Nagelkerke *R*^2^ = 0.41, 80.0% predicted correctly)					
Population	−4.06	1.81	5.06	0.024	0.02 (0.00–0.59)
Snake length	0.11	0.10	1.21	0.272	1.12 (0.92–1.35)
Duration captivity	−0.01	0.06	0.04	0.837	0.99 (0.88–1.11)

^a^ Snake size and duration in captivity were treated as continuous variables.

**Table 3:** Analysis of covariance (ANCOVA) models for quantitative antipredator behaviors of mainland (*N* = 20) and Santa Catalina Island (*N* = 10), California, USA, populations of the southern Pacific rattlesnake (*Crotalus helleri*).

Variables ^a^	*F*-Value ^b^	*p*-Value	Partial η^2^
Latency to capture (s)			
Population	16.62	<0.001	0.39
Snake length	0.88	0.358	0.03
Duration captivity	0.33	0.573	0.01
Venom expended (mg)			
Population	11.64	0.002	0.33
Snake length	13.62	0.001	0.36
Duration captivity	0.54	0.472	0.02
Bilateral venom pulses (number)			
Population	1.99	0.172	0.08
Snake length	0.04	0.843	0.00
Duration captivity	0.00	0.994	0.00
Venom per bite (mg)			
Population	7.31	0.012	0.23
Snake length	12.07	0.002	0.34
Duration captivity	0.01	0.922	0.00

^a^ One snake refused to deliver venom; thus, for the three venom variables, *N* = 19 and 9 for mainland and Santa Catalina Island populations, respectively. ^b^ df = 1.26 for the latency to capture model, and df = 1.24 for the three venom models.

**Table 4:** Potential mammalian predators (cat, fox, and pig) and antagonists (herbivorous ungulates) of rattlesnakes on Santa Catalina Island (194 km^2^), California, USA, including dates for introduction to and removal from the island, and highest historical population estimates (*N*) and density (island-wide *N*/km^2^). ^a^ All are introduced species with the possible exception of the fox.

Species	Introduction	Removal	Highest *N*	Highest Density	Distribution	Source
Cat (*Felis catus*)	1800s	extant	600–750	3.1–3.9	island-wide	[69]
Island fox (*Urocyon littoralis catalinae*)	7100–9200 years ago	extant	1115–1342	5.7–6.9	island-wide	[70,71,72]
Pig (*Sus scrofa*)	early 1930s	2004	1260–5000	6.5–25.8	island-wide	[73,74,75,76]
American bison (*Bison bison*)	1924	extant	400–524	2.1–2.7	currently east end	[76,77,78,79]
Black buck (*Antilope cervicapra*)	1967–1973 (uncertain)	2014	<25	~0.1	east end	[80,81,82]
Cattle (*Bos taurus*)	1800s	1950s	>5225	26.9	island-wide	[76,78,83]
Goat (*Capra hircus*)	early 1800s	2003	30,000–50,000	154.6–257.7	island-wide	[76,84,85,86]
Horse (*Equus caballus*)	1800s	unknown	240	1.2	unknown	[76]
Mule deer (*Odocoileus hemionus*)	1928	extant	2000–3285	5.4–16.9	island-wide	[76,87,88]
Sheep (*Ovis aries*)	1800s	1920s	22,000	113.4	island-wide	[76,78,86]

^a^ Domestic dogs (*Canis lupus familiaris*) were likely common at one time [62], but currently exist in human residential areas without a substantial feral population.

## Data Availability

Data can be made available upon request.

## Institutional Animal Care and Use Statement

Research was conducted under protocols 88059 and 8110041 approved by the Loma Linda University Institutional Animal Care and Use Committee (approval date: 1 November 2011).
